# Identification of hsa_circ_0001821 as a Novel Diagnostic Biomarker in Gastric Cancer *via* Comprehensive Circular RNA Profiling

**DOI:** 10.3389/fgene.2019.00878

**Published:** 2019-09-20

**Authors:** Shan Kong, Qian Yang, Chenxue Tang, Tianyi Wang, Xianjuan Shen, Shaoqing Ju

**Affiliations:** ^1^Department of Laboratory Medicine, Affiliated Hospital of Nantong University, Nantong, China; ^2^Research Center of Clinical Medicine, Affiliated Hospital of Nantong University, Nantong, China

**Keywords:** circular RNA, gastric cancer, high-throughput sequencing, biomarker, diagnosis

## Abstract

**Background:** The morbidity and mortality of gastric cancer (GC) remain high worldwide. With the advent of the Human Genome Sequencing Project, circular RNAs (circRNAs) have attracted widespread attention in cancer research due to their stable ring structure. Our aim was to identify differentially expressed circRNAs in GC and explore their potential roles in GC diagnosis, treatment, and prognostic prediction.

**Methods:** Large-scale gene screening was performed in three pairs of GC tissues and adjacent noncancerous tissues using high-throughput sequencing. The expression of hsa_circ_0001821 was detected in 80 pairs of tissue samples by quantitative real-time PCR (qRT-PCR). Stability of the ring structure of hsa_circ_0001821 RNA was verified by exonuclease digestion assay, and its diagnostic value was evaluated by receiver operating characteristic (ROC) analysis. In addition, the location of hsa_circ_0001821 in GC cells was detected by nucleoplasm separation assay.

**Results:** A total of 25,303 circRNAs were identified, among which 2,007 circRNAs were differentially expressed (fold change > 2.0, *P* < 0.05). Further validation disclosed that hsa_circ_0001821 was significantly downregulated in the 80 pairs of GC tissues and 30 whole-blood specimens obtained from the GC patients. The specificity of hsa_circ_0001821 in GC was higher than that in other solid tumors. In addition, hsa_circ_0001821 was relatively stable after RNA exonuclease digestion. Clinicopathological parameter analysis showed that hsa_circ_0001821 was negatively correlated with tumor depth (*r* = −0.255, *P* = 0.022) and lymph node metastasis (*r* = −0.235, *P* = 0.036). Area under the curve (AUC) analysis showed that the diagnostic efficiency of circulating hsa_circ_0001821 in distinguishing GC patients was higher than that in GC tissues (0.872, 95%CI: 0.767–0.977 *vs.* 0.792, 95%CI: 0.723–0.861). Combined use of circulating hsa_circ_0001821 with the existing tumor markers yielded the largest AUC of 0.933. Finally, hsa_circ_0001821 was demonstrated to mainly locate in the cytoplasm, implying that it played a potential regulatory role in GC at the posttranscriptional level.

**Conclusion:** Hsa_circ_0001821 may prove to be a new and promising potential biomarker for GC diagnosis.

## Introduction

Gastric cancer (GC) remains one of the most common malignant tumors worldwide. According to the latest statistics released by the World Health Organization (WHO) Cancer Control Program, over seven million people die of cancer worldwide each year, with about 700,000 of them suffering from GC (Siegel et al., 2019). Meanwhile, approximately 934,000 new cases of GC are diagnosed every year, among which about 43% (400,000) occur in China with morbidity and mortality rates about twofold higher than the world average (Cheng et al., 2016; Miller et al., 2016). Usually, patients with advanced GC may have a 50–70% chance of recurrence after surgery, and their 5-year survival rate is often less than 30% (Sun and Yan, 2016). Currently, the early detection rate of GC is less than 10%, and the disease is usually diagnosed in the advanced stage or when metastasis has already occurred. Therefore, it is of particular significance to screen out specific and sensitive biomarkers and strengthen the research on GC pathogenesis for the sake of improving the diagnosis and treatment of GC.

Using the Human Genome Sequencing Project, scientists have found that the proportion of protein-coding genes in the transcriptome is much lower than that in noncoding RNAs (ncRNAs), and about 80% transcription products are ncRNAs (Prasanth and Spector, 2007). Initially, most ncRNAs were considered to be the “noise” of genome transcription and therefore largely ignored. However, with the reduction of sequencing cost and the emerging of new-generation sequencing technology and in-depth sequencing of complementary DNA (cDNA) pools or libraries, ncRNAs have been identified to act as regulatory factors to control gene expression at multiple cell levels and maintain telomere elongation. Meanwhile, they are viewed as guides of molecular repair with important biological functions in life activities and disease occurrence (Taft et al., 2010). Presently, three specific ncRNAs are widely reported in cancer research, including microRNAs (miRNAs), long noncoding RNAs (lncRNAs) > 200 nt, and newly discovered circular RNAs (circRNAs) (Beermann et al., 2016).

circRNAs are a group of endogenous ncRNA molecules that widely exist in human cells. Current studies have demonstrated that circRNA is produced by special variable shear, and its 3′ and 5′ ends are joined together by covalent bonding to form a closed circular structure. Compared with other types of ncRNAs, circRNA is well tolerable by RNA exonuclease, relatively stable, and not easily degradable, making it a highly variable competitive endogenous RNA (ceRNA) (Lasda and Parker, 2014). A single circRNA molecule contains a large number of miRNA response elements that can bind or release a large number of miRNAs instantaneously, so circRNA acts very efficiently and stably as a ceRNA (Qu et al., 2015). Evidence shows that circRNAs can regulate gene expression at the transcriptional level *via* binding miRNAs as a molecule sponge. On the other hand, circRNAs might bind to RNA-binding proteins or other RNA translation proteins through complementary base pairs, interfering with the normal function of genes at the posttranscriptional level. These findings provide a new direction for the exploration of circRNAs as targets for disease diagnosis and prognostic prediction.

To find differentially expressed circRNAs, we detected circRNA expression in three pairs of GC tissues by high-throughput sequencing in the present study and identified 2,007 significantly differentially expressed circRNAs *via* circRNA sequencing. Subsequently, we chose hsa_circ_0001821 as our study object to further our investigation in 80 pairs of GC tissues and 30 whole-blood samples from GC patients and evaluate the clinical utility of hsa_circ_0001821 in GC diagnosis by receiver operating characteristic (ROC) analysis in an attempt to provide a novel biomarker for GC research.

## Results

### Identification of Deregulated circRNAs in GC Tissues

**Figure 1:**
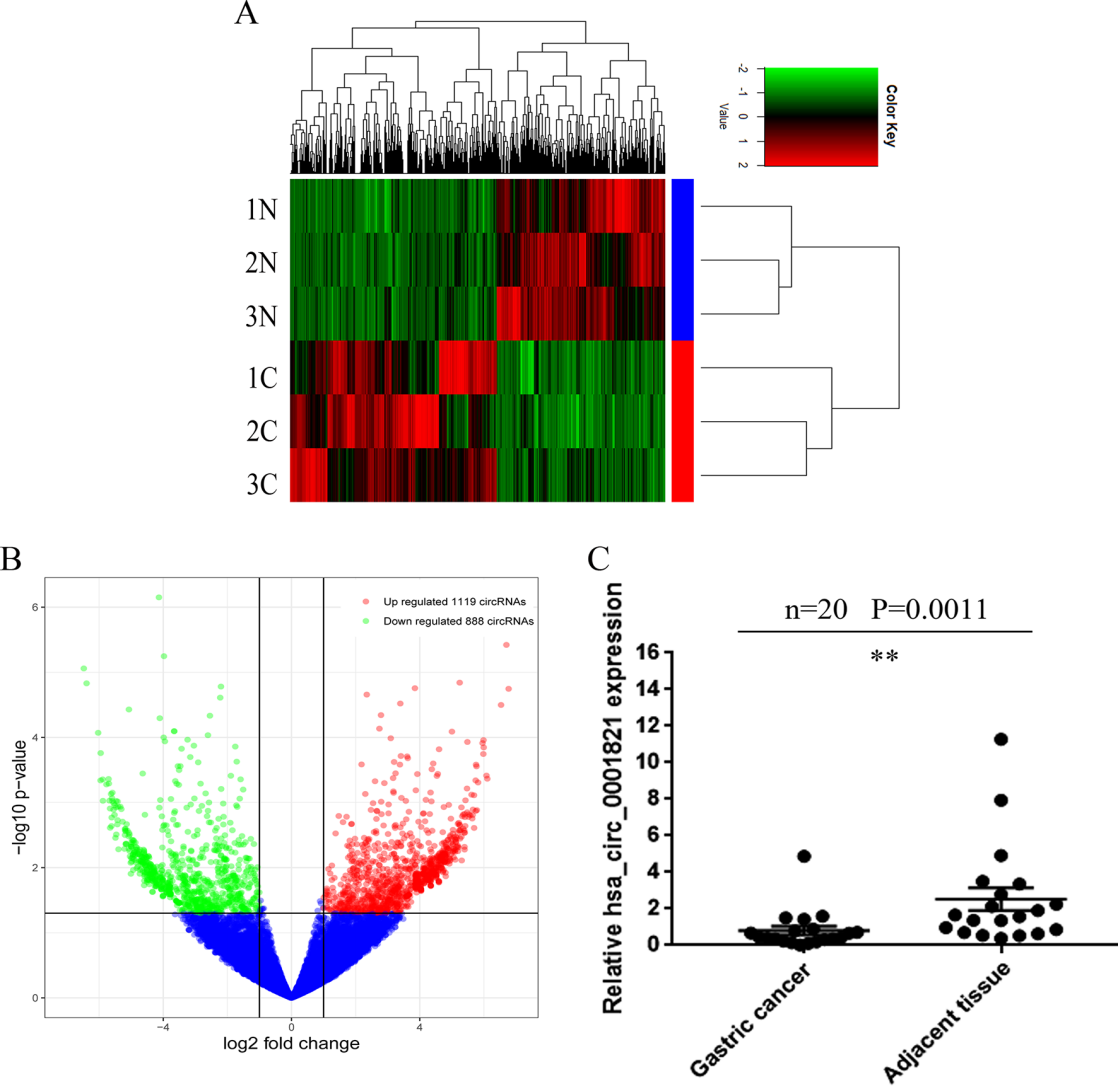
Identification of deregulated circular RNAs (circRNAs) in gastric cancer (GC) tissues. **(A)** Clustered heatmap. Each row represents a tissue sample, and each column represents a circRNA. The color scale reflects the log_2_ signal strength from green (low intensity) to black (medium intensity) to red (strong intensity). **(B)** Volcano plots. The red points in plot indicate the differentially upregulated expression of circRNAs with statistical significance while the green points indicate the downregulated circRNAs. **(C)** Initial verification of hsa_circ_0001821 expression in 20 pairs of GC tissues by quantitative real-time PCR (qRT-PCR). **P<0.01 were considered significant.

To investigate the expression profiles of circRNAs in GC tissues, we conducted high-throughput sequencing in three GC tissues *vs.* three matched noncancerous tissues and identified a total of 25,303 circRNA targets, including 20,036 known circRNAs and 5,267 undefined circRNAs. The heatmap was depicted as a direct approach to visualize the distributions of the dataset for circRNA profiles (**Figure 1A**). Volcano plots depicted 2,007 differentially expressed circRNAs in the GC tissues (fold change > 2.0, *P* < 0.5) (**Figure 1B**), from which 16 significantly different circRNAs were selected (2.0 < fold change < 6.0, *P* < 0.05). The details regarding these circRNAs are presented in **Table 1**. Knowing that the specific parental gene is a lncRNA closely associated with GC evolution and progression (Xu et al., 2017; Zhao et al., 2018), we finally chose hsa_circ_0001821 as our research target. We first detected hsa_circ_0001821 expression in 20 pairs of GC tissues by quantitative real-time PCR (qRT-PCR) and found that it was significantly downregulated in the GC tissues (**Figure 1C**).

**Table 1:** A total of 16 significantly differentially expressed circRNAs identified *via* circRNA sequencing.

circRNA	Gene	Fold change	*P value*	Chr	Type
hsa_circ_0001364	GNB4	2.251337211	0.033633989	3	Exons
hsa_circ_0001821	PVT1	3.287035969	0.001654397	8	Exons
hsa_circ_0041638	ENO3	4.89323537	0.008760362	17	Exons
hsa_circ_0012397	FAF1	3.060338579	0.046433022	1	Exons
hsa_circ_0000665	UBE2I	5.659752071	0.042535215	16	Exons
hsa_circ_0009109	DCAF6	2.800439031	0.039049502	1	Exons
hsa_circ_0004119	RAB23	4.324163375	0.008493806	6	Exons
hsa_circ_0008192	PTBP3	3.677016974	0.002126932	9	Exons
hsa_circ_0001998	FUT8	-3.222198465	0.018780302	14	Exons
hsa_circ_0069338	SEPSECS	-2.876947158	0.018265975	4	Exons
hsa_circ_0015262	SUCO	-2.506859008	0.020001902	1	Exons
hsa_circ_0006559	TXNDC11	-5.320530101	0.005367026	16	Exons
hsa_circ_0001818	UBR5	-3.361273221	0.020285073	8	Exons
hsa_circ_0094976	ALG9	-4.805587534	0.041057838	11	Exons
hsa_circ_0063809	CELSR1	-4.065924801	0.007520253	22	Exons
hsa_circ_0001766	PDIA4	-2.841537714	0.000632629	7	Exons

### Methodological Evaluation of hsa_circ_0001821 in GC Cells

**Figure 2:**
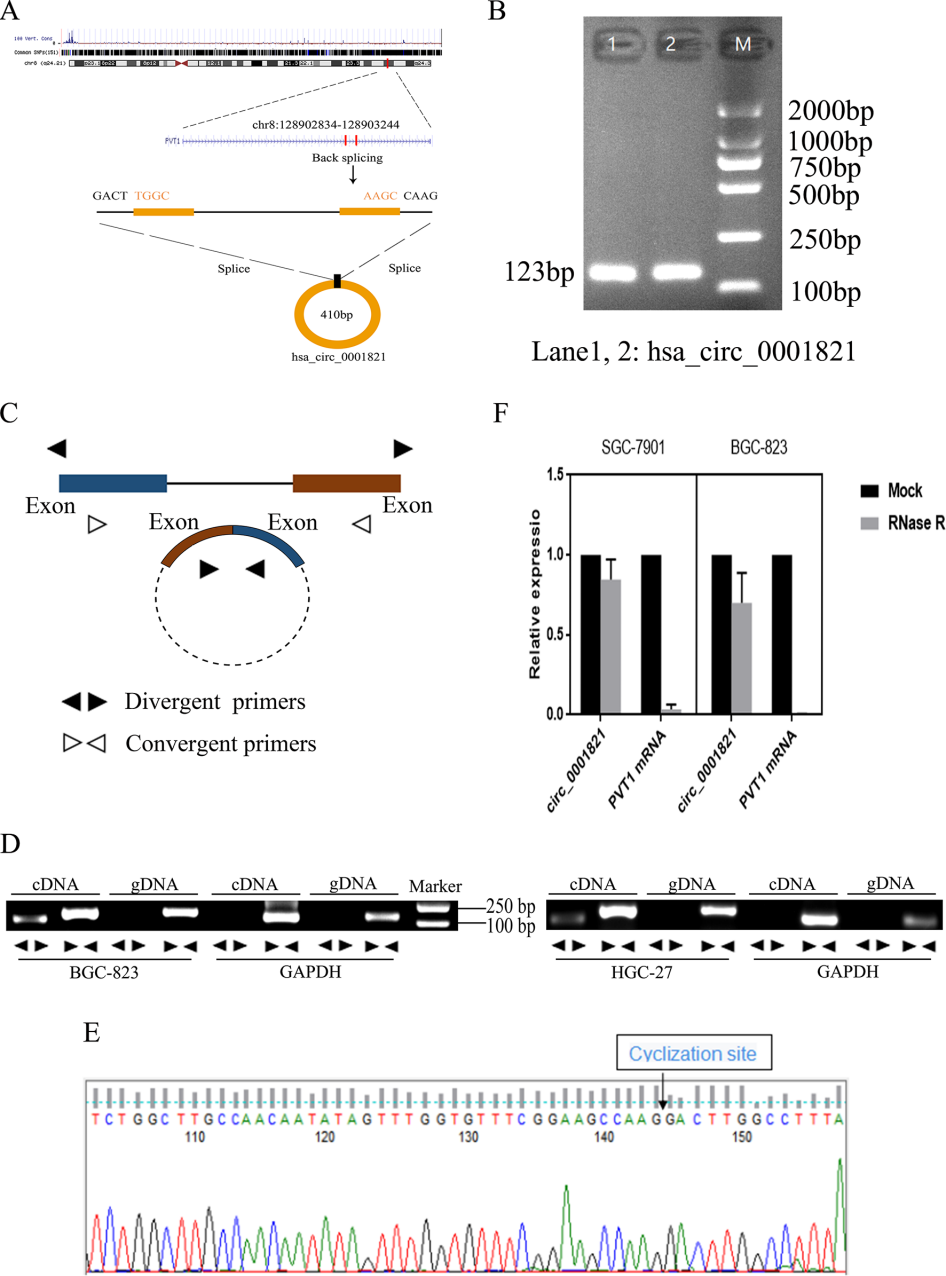
Methodological evaluation of hsa_circ_0001821 in gastric cancer (GC) cells. **(A)** The origin of hsa_circ_0001821 searched through the University of California Santa Cruz (UCSC) genome browser. **(B)** Verification of the size of the primer amplification product (123 bp) by agarose gel electrophoresis. **(C)** A schematic diagram for the design of polymerized primers and reverse primers. **(D)** Verification of the ring structure of hsa_circ_0001821. **(E)** Detection of the cyclization site by Sanger sequencing. **(F)** Hsa_circ_0001821 was tolerable to the degradation of RNA exonuclease in SGC-7901 and BGC-823 cells.

According to the human reference genome (GRCh37/hg19) from the Ensembl genome database (http://www.ensembl.org), hsa_circ_0001821 is located at chr8_128902834_128903244_+, and the length of its mature transcript is 410 bp (**Figure 2A**). To verify the specificity and accuracy of the amplification procedure, the PCR amplification products were subjected to 2.5% agarose gel electrophoresis. The single electrophoresis bands were consistent with the size of the primer amplification product (**Figure 2B**). To verify the ring structure of hsa_circ_0001821, we designed polymerized primers and reverse primers for their cyclization sites (**Figure 2C**). qRT-PCR was then performed using genomic DNA (gDNA) and cDNA as templates and glyceraldehyde 3-phosphate dehydrogenase (GAPDH) as the negative control. Agarose electrophoresis assay showed that hsa_circ_0001821 could be amplified from the PCR products using cDNA as the template, while a negative result was observed in the control group using gDNA as the template (**Figure 2D**). Besides, the reverse shear site of hsa_circ_0001821 was confirmed by Sanger sequencing (**Figure 2E**). Knowing that circRNA is relatively stable compared with linear RNA and not easily degraded by RNA exonuclease, we performed the RNA exonuclease digestion assay. RNA exonuclease was added to total RNA isolated from SGC-7901 and BGC-823 cells, and the expression of hsa_circ_0001821 and linear PVT1 was detected by qRT-PCR. Compared with that of linear PVT1, hsa_circ_0001821 expression was not significantly reduced after RNA exonuclease treatment, indicating that hsa_circ_0001821 had a relatively stable structure (**Figure 2F**).

### Correlation Analysis of hsa_circ_0001821 Expression and the Clinicopathological Parameters in GC Patients

As shown in **Table 2**, the expression of hsa_circ_0001821 in GC tissues was significantly correlated with tumor depth (*P* = 0.0030) and lymph node metastasis (*P* = 0.0072). However, we did not find any association between the hsa_circ_0001821 expression and other clinicopathological parameters, such as gender (*P* = 0.8285), age (*P* = 0.1887), histological differentiation (*P* = 0.0696), tumor size (*P* = 0.8900), CEA (*P* = 0.0977), CA199 (*P* = 0.0864), and CA125 (*P* = 0.7259). Furthermore, the Spearman correlation analysis also indicated that hsa_circ_0001821 expression was negatively correlated with tumor depth (*r* = −0.255, *P* = 0.022) and lymph node metastasis (*r* = −0.235, *P* = 0.036) (**Table 3**).

**Table 2:** The association between hsa_circ_0001821 expression and the clinicopathological parameters in GC patients.

Characteristics	n	High expression (n=18)	Low expression (n=62)	P-Value
Gender				
male	55	12	43	0.8285
female	25	6	19	
Age(years)				
≥60	62	16	46	0.1887
<60	18	2	16	
Histological differentiation				
Poorly	46	7	39	0.0696
Moderately	34	11	23	
Tumor depth				
T1-T2	19	9	10	0.0030**
T3-T4	61	9	52	
Lymph node metastasis				
Yes	56	8	48	0.0072**
No	24	10	14	
Tumor size(cm)				
≥5	30	7	23	0.8900
<5	50	11	39	
CEA(ng/ml)				
>5.0	27	9	18	0.0977
≤5.0	53	9	44	
CA199(U/ml)				
>37.0	19	7	12	0.0864
≤37.0	61	11	50	
CA125(U/ml)				
>35.0	24	6	18	0.7259
≤35.0	56	12	44	

Statistical analyses were carried out using Pearson χ2 test.

**P<0.01 was considered significant.

**Table 3:** Spearman correlation analysis of hsa_circ_0001821 expression and the clinicopathological parameters in GC patients.

Variables	hsa_circ_0001821 expression level	
	Spearman correlation	P-Value
Gender	-0.046	0.684
Age(years)	0.022	0.846
Histological differentiation	0.010	0.927
Tumor depth	-0.255	0.022*
Lymph node metastasis	-0.235	0.036*
Tumor size(cm)	0.004	0.969
CEA(ng/ml)	-0.177	0.116
CA199(U/ml)	-0.193	0.087
CA125(U/ml)	-0.045	0.689

*P<0.05 was considered significant.

### Validation of hsa_circ_0001821 Expression in Different Tumor Tissues

**Figure 3:**
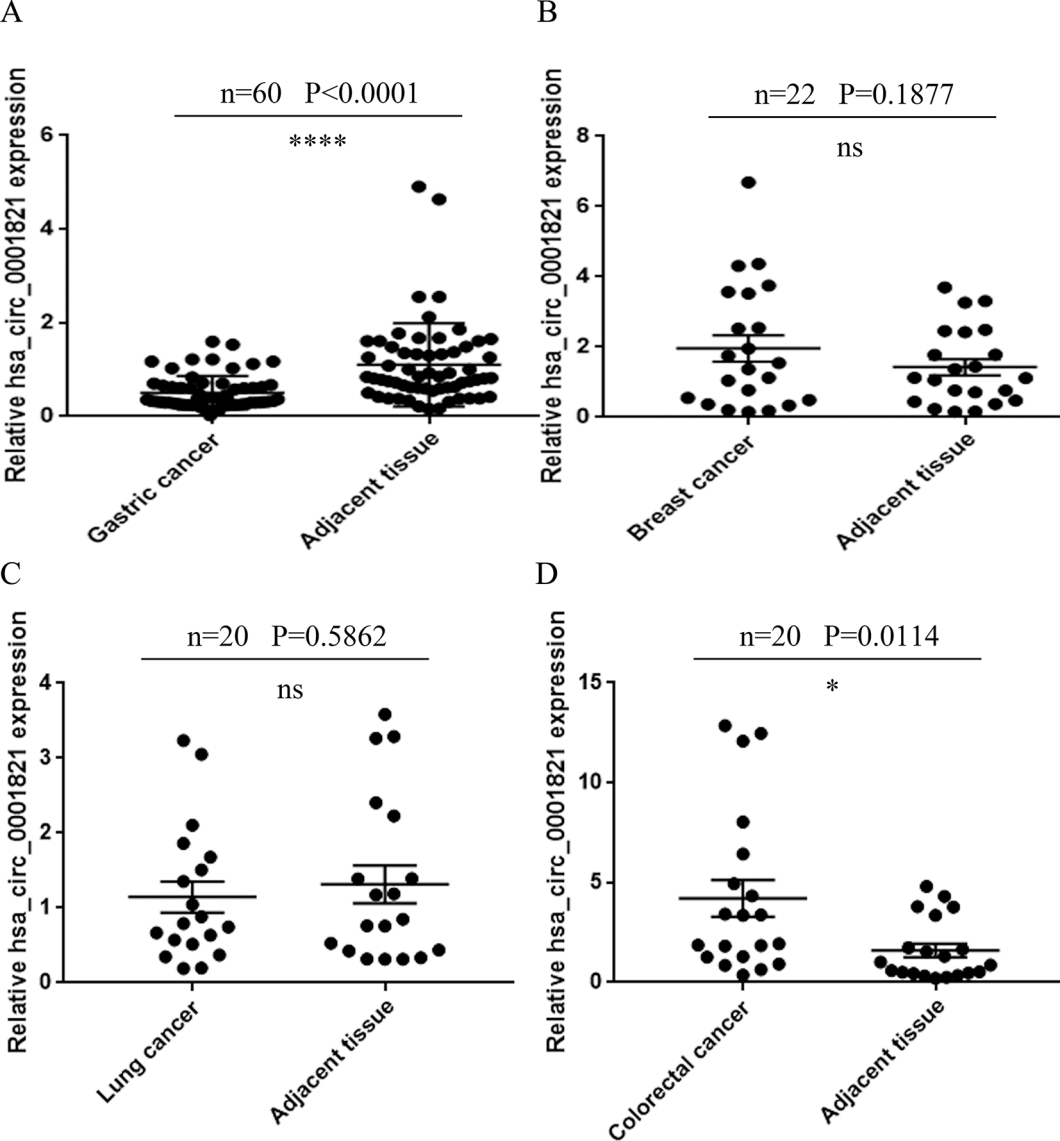
Validation of hsa_circ_0001821 expression in different tumor tissues. **(A)** Extended verification of hsa_circ_0001821 expression in 60 pairs of gastric cancer (GC) tissues by quantitative real-time PCR (qRT-PCR). Detection of hsa_circ_0001821 expression in breast cancer (**B**, *n* = 22), lung cancer (**C**, *n* = 20), and colorectal cancer tissues (**D**, *n* = 20). *P<0.05 and ****P<0.0001 were considered significant.

For large-sample verification, 60 pairs of GC tissues were collected. The result of qRT-PCR showed that the expression of hsa_circ_0001821 in GC tissues was significantly lower than that in adjacent normal tissues (*P* < 0.0001) (**Figure 3A**). To verify the organ specificity of hsa_circ_0001821, the relative expression of hsa_circ_0001821 was calculated in 20 pairs of breast cancer tissues (**Figure 3B**), 22 pairs of lung cancer tissues (**Figure 3C**), and 20 pairs of colorectal cancer (CRC) tissues (**Figure 3D**). The results showed that the hsa_circ_0001821 expression in breast cancer and lung cancer was not statistically significant while it was increased in CRC tissues. With the clinicopathological parameters of these patients taken into account, we may conclude that hsa_circ_0001821 was organ specific in GC.

### Evaluation of the Diagnostic Value of hsa_circ_0001821 in GC Patients

**Figure 4:**
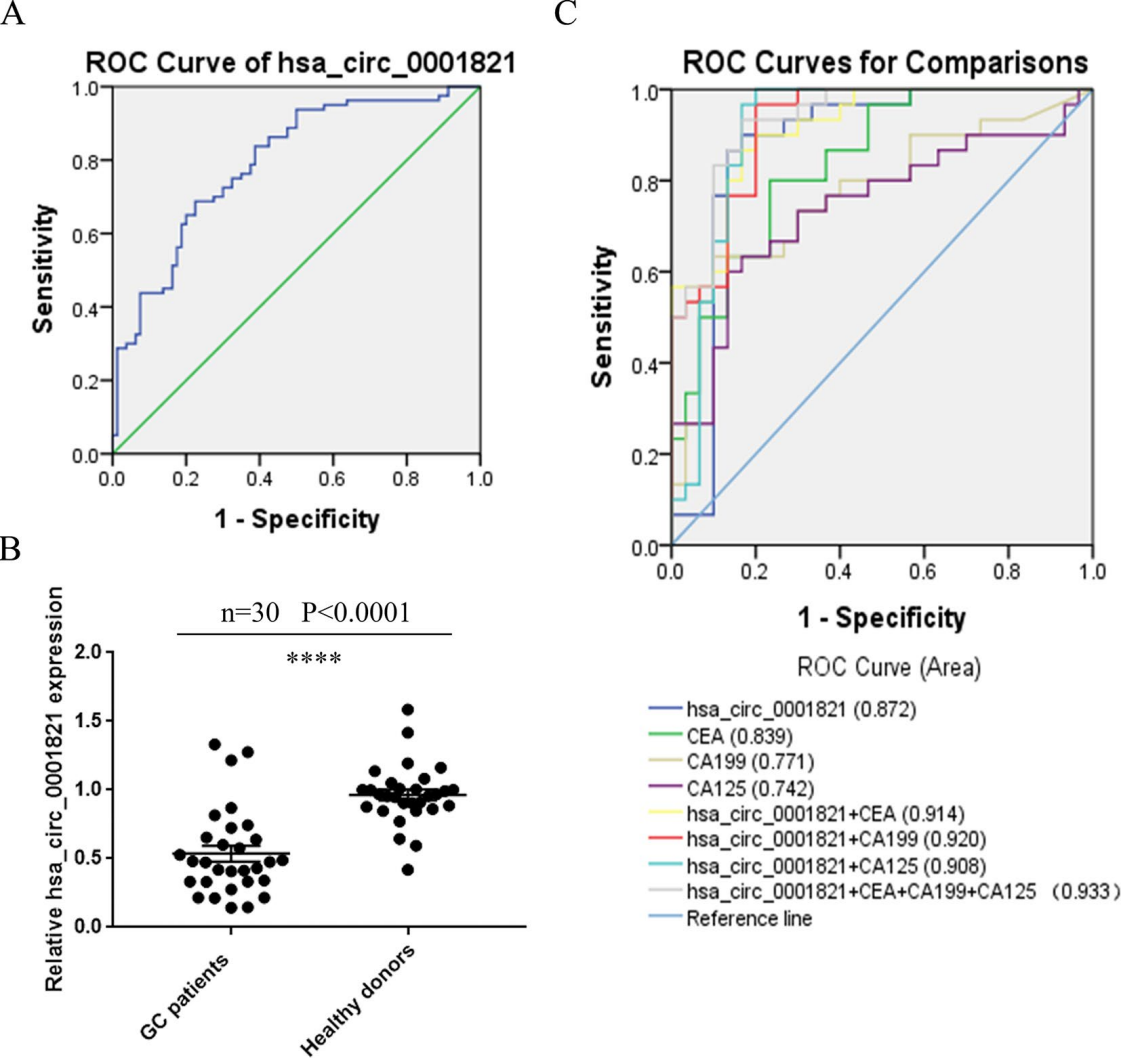
Evaluation of the diagnostic value of hsa_circ_0001821 in gastric cancer (GC) patients. **(A)** Receiver operating characteristic (ROC) analysis of hsa_circ_0001821 in differentiating GC tissues from noncancerous tissues (*n* = 80). **(B)** Detection of circulating hsa_circ_0001821 expression in the whole-blood samples from GC patients (*n* = 30) and healthy donors (*n* = 30). **(C)** The construction of the joint diagnostic model containing circulating hsa_circ_0001821 and existing laboratory indicators. ****P<0.0001 was considered significant.

To see whether hsa_circ_0001821 could be utilized as a potential GC diagnostic marker, we depicted the ROC curve and calculated the area under the curve (AUC) based on the data obtained from the 80 pairs of GC tissues. The AUC of hsa_circ_0001821 in differentiating GC tissues from noncancerous ones was 0.792 (95%CI: 0.723–0.861, *P* < 0.001) (**Figure 4A**). In view of the noninvasiveness of liquid biopsy, we also detected the expression of hsa_circ_0001821 in peripheral blood samples of 30 GC patients and collected 30 fresh normal whole-blood samples as the healthy control. Consistent with the finding in the tissue samples, the hsa_circ_0001821 expression was also downregulated in the peripheral blood samples of GC patients (**Figure 4B**). Then we performed ROC analysis to verify the clinical utility of circulating hsa_circ_0001821 in GC diagnosis. The data showed that the AUC of circulating hsa_circ_0001821 in distinguishing GC patients from the healthy donors was 0.872 (95%CI: 0.767–0.977), which is higher than that of CEA (0.839, 95%CI: 0.740–0.937), CA199 (0.771, 95%CI: 0.649–0.893), and CA125 (0.742, 95%CI: 0.613–0.871) (**Figure 4C**). More importantly, the combined use of circulating hsa_circ_0001821 and the existing tumor markers CEA, CA199, and CA125 yielded the largest AUC of 0.933 (**Figure 4C**). Statistical analysis also showed that the combination of circulating hsa_circ_0001821 and CA199 significantly provided a sensitivity of 93.33% (**Table 4**).

**Table 4:** Evaluation of the diagnostic values of combination of hsa_circ_0001821, CEA, CA199 and CA125.

	SEN, %	SPE, %	ACCU, %	PPV, %	NPV, %
hsa_circ_0001821	86.67(26/30)	86.67(26/30)	86.67(52/60)	86.67(26/30)	86.67(26/30)
CEA	63.33(19/30)	86.67(26/30)	75.00(45/60)	82.61(19/23)	70.27(26/37)
CA199	60.00(18/30)	90.00(27/30)	75.00(45/60)	85.71(18/21)	69.23(27/39)
CA125	60.00(18/30)	86.67(26/30)	73.33(44/60)	81.82(18/22)	68.42(26/38)
hsa_circ_0001821+CEA	90.00(27/30)	80.00(24/30)	85.00(51/60)	81.82(27/33)	88.89(24/27)
hsa_circ_0001821+CA199	93.33(28/30)	80.00(24/30)	86.67(52/60)	82.35(28/34)	92.31(24/26)
hsa_circ_0001821+CA125	90.00(27/30)	73.33(22/30)	81.67(49/60)	77.14(27/35)	88.00(22/25)
hsa_circ_0001821+CEA +CA199+CA125	93.33(28/30)	63.33(19/30)	78.33(47/60)	71.79(28/39)	90.48(19/21)

SEN, sensitivity; SPE, specificity; ACCU, overall accuracy; PPV, positive predictive value; NPV, negative predictive value.

### Exploration of the Downstream Regulatory Network of hsa_circ_0001821 in GC Cells

**Figure 5:**
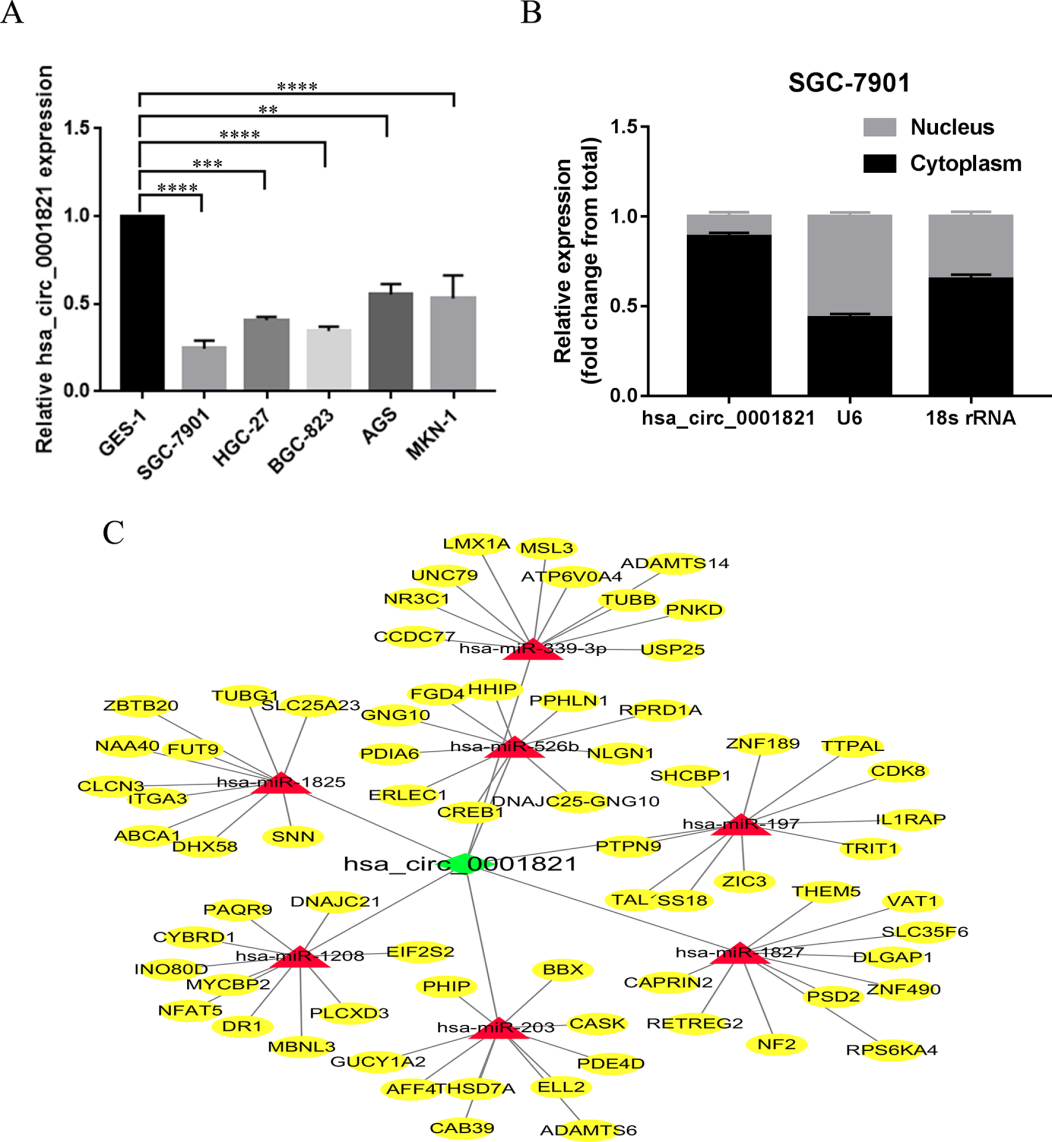
Exploration of the downstream regulatory network of hsa_circ_0001821 in gastric cancer (GC) cells. **(A)** Detection of hsa_circ_0001821 expression in five GC cell lines. **(B)** Detection of hsa_circ_0001821 location in SGC-7901 cell line by nucleoplasm separation assay. **(C)** Prediction of circular RNA (circRNA)-microRNA (miRNA)–messenger RNA (mRNA) network map of hsa_circ_0001821. The green diamond represents hsa_circ_0001821, and the red rectangle represents seven miRNAs that could interact with hsa_circ_0001821, while the yellow oval represents the target mRNA of the corresponding miRNA.

To investigate the functional mechanism of hsa_circ_0001821 in GC cells, the expression levels of hsa_circ_0001821 in five GC cell lines (SGC-7901, HGC-27, BGC-823, AGS, and MKN-1) were detected, using the normal gastric mucosal epithelial GES-1 cells as the control. Similarly, hsa_circ_0001821 showed a significantly lower expression level in the five GC cell lines (*P* < 0.01) (**Figure 5A**). We further extracted RNA from SGC-7901 cells by nucleoplasm separation and found that hsa_circ_0001821 accounted for a higher proportion in the cytoplasm, suggesting that it might participate in GC progression mainly through posttranscriptional regulation (**Figure 5B**). Next, the potential circRNA–miRNA–mRNA regulatory axis in GC was predicted by using high-throughput sequencing and bioinformatics analysis. As shown in **Figure 5C**, seven miRNAs (miR-1208, miR-1825, miR-197, miR-203, miR-339-3p, miR-526b, and miR-1827) and their corresponding target mRNAs were depicted, which may provide a new direction in exploring the regulatory network of hsa_circ_0001821 in GC in the future.

## Discussion

circRNAs are a subclass of ncRNAs widely expressed in mammalian cells. Ample evidence has shown that circRNAs are mainly produced by precursor messenger RNAs (pre-mRNAs) *via* variable splicing (Ashwal-Fluss et al., 2014). circRNAs were first detected in RNA viruses in the 1970s (Kos et al., 1986). Then in 1979, researchers discovered for the first time under the electron microscope that circRNAs were also present in the cytoplasm of eukaryotic cells (Hsu and Coca-Prados, 1979). It was also reported that some exon-derived circRNAs existed in the mitochondria of yeast and human cells (Arnberg et al., 1980). However, due to the immature technology at that time, circRNAs were only regarded as a kind of low-abundance RNA molecule formed by the incorrect splicing of exon transcripts and were not further studied. With the development of high-throughput sequencing technology and bioinformatics, circRNAs have been widely found in eukaryotic cells, and their expression levels are specific to species, tissues, and time (Memczak et al., 2013).

More studies have reported that circRNAs are involved in the development of malignant tumors. A study revealed that a group of circRNAs specifically participated in the invasive growth of pancreatic ductal adenocarcinoma cells (Li et al., 2018b). Besides, a series of circRNAs has been reported to be aberrantly expressed in hepatocellular carcinoma (HCC), making early detection of liver cancer possible (Zhang et al., 2018). In our study, we used three pairs of fresh GC tissues and their corresponding benign adjacent tissues to identify a number of circRNAs with significant expression differences through circRNA-seq. From the 2,007 differentially expressed circRNAs, we selected 16 circRNAs for initial verification, among which we finally selected hsa_circ_0001821 in view of the relationship between its parental genes and GC. Our present study showed that hsa_circ_0001821 was significantly downregulated in both GC tissues and whole-blood specimens, implying the potential role of hsa_circ_0001821 in GC evolution. However, one contradiction was that the expression trend of hsa_circ_0001821 in GC tissues *via* high-throughput sequencing was contrary to that detected by qRT-PCR. A similar situation also appeared in Li’s article (Li et al., 2018a). After a simple analysis, we suspected that the small sample size and individual differences might be the main factors accounting for the contradictory results. High-throughput sequencing consisted of only three pairs of GC tissues, which may not represent the total number of GC patients, and there might be individual differences between each GC patient.

An ideal tumor marker should be organ specific. Our study discovered that hsa_circ_0001821 was not significantly expressed in breast cancer and lung cancer tissues but upregulated in CRC tissues, which is opposite to the finding in GC tissues. Analysis on the clinicopathological parameters also showed that hsa_circ_0001821 was significantly correlated with tumor depth and lymph node metastasis of GC patients. Spearman correlation analysis also indicated that decreased hsa_circ_0001821 expression was negatively correlated with tumor depth and lymph node metastasis. However, no correlation was observed between hsa_circ_0001821 and other tumors, supporting its organ specificity in GC. Knowing that circRNAs are mainly present in white blood cells (WBCs) but are excessively depleted in the serum samples, we chose whole-blood samples to isolate circulating circRNAs. ROC analysis proved that the AUC of circulating hsa_circ_0001821 in distinguishing GC patients from the healthy donors was 0.872, which is higher than that in GC tissues and other laboratory markers of CEA, CA199, and CA125. More importantly, combining circulating hsa_circ_0001821 with other existing tumor markers yielded a maximum AUC of 0.933. These results suggest that hsa_circ_0001821 could be utilized as a biomarker with favorable sensitivity and specificity in GC.

As circRNAs are connected at the 3′ and 5′ ends by exon or intron cyclization forming a complete ring structure, they are not easily degraded by exonuclease and therefore more stable than linear RNAs (Lasda and Parker, 2014). It was found in our study that hsa_circ_0001821 was not significantly degraded after RNA exonuclease treatment as compared with linear PVT1, indicating that hsa_circ_0001821 is relatively stable. Evidence has shown that increased lncRNA PVT1 expression is closely correlated with GC progression. For example, PVT1 was reported to participate in angiogenesis *via* activating the STAT3/VEGFA axis in GC (Zhao et al., 2018). Besides, it was highly responsible for cisplatin resistance and multidrug resistance in GC cells (Zhang et al., 2015; Zhang et al., 2017). Other reports showed that PVT1 might serve as a promising biomarker for early detection and prognostic prediction of GC (Kong et al., 2015; Yuan et al., 2016). It was found in our study that hsa_circ_0001821 originating from its parent gene PVT1 (which was upregulated in GC) had a more stable ring structure and was significantly downregulated in GC. This different expression trend between hsa_circ_0001821 and its parental gene PVT1 will inspire us to explore its regulatory axis in our future research.

In view of the characteristics and their increasing importance in tumor development, we believed that circRNAs have advantages in acting as clinical diagnostic markers, and we hope that further study on the circRNA-associated mechanism in GC development would shed new light on GC treatment. It was found in our study that hsa_circ_0001821 was significantly downregulated in the five GC cell lines. Our nucleoplasm separation assay indicated that hsa_circ_0001821 accounted for a higher proportion in the cytoplasm, suggesting that it may play a regulatory role in GC progression at the posttranscriptional level. Additionally, the circRNA–miRNA–mRNA regulatory axis in GC was predicted. The bioinformatics analysis illustrated that hsa_circ_0001821 could potentially interact with miR-1208, miR-1825, miR-197, miR-203, miR-339-3p, miR-526b, and miR-1827. Among these miRNAs, miR-197 was found to exert an inhibitory effect on human gastric carcinogenesis and progression by regulating the MTDH/PTEN/AKT signaling pathway (Liao et al., 2018). Besides, miR-203 was able to inhibit the malignant phenotype of GC cells and served as a noninvasive biomarker for predicting prognosis and metastasis in GC patients (Imaoka et al., 2016; Zhou et al., 2016; Gao et al., 2017; Li et al., 2019). The rs8506G > a polymorphism at the miR-526b binding site was responsible for noncardia GC risk (Fan et al., 2014). These findings reveal a diverse regulatory network in GC, in which hsa_circ_0001821 might be involved.

In summary, we identified approximately 2,007 circRNAs that were significantly differentially expressed in GC through high-throughput sequencing. Among these circRNAs, hsa_circ_0001821 was significantly downregulated in both GC tissues and whole-blood specimens. These data suggest that hsa_circ_0001821 may prove to be a potential diagnostic biomarker of GC. The combination of hsa_circ_0001821 with existing immunohistochemical markers could significantly improve the diagnostic accuracy. But as the present study is a preinvestigational study, the detailed mechanism of hsa_circ_0001821 in GC remains to be confirmed, and the circRNA–miRNA–mRNA regulatory axis predicted by bioinformatics needs to be further verified in a future study so as to improve our understanding about the role of hsa_circ_0001821 in GC progression.

## Materials and Methods

### Specimen Collection

From September 2016 to December 2018, 80 pairs of GC tissues were collected in the Affiliated Hospital of Nantong University (Nantong, China). The tissue samples were added to an RNA fixative agent (Bioteke, Beijing, China) immediately after excision and stored at −80°C. In addition, a total of 60 peripheral blood samples (stored in EDTA tubes), including 30 GC patients and 30 healthy controls, were also included in this study. All the included patients were diagnosed by professional pathologists and clinicians and did not receive preoperative chemotherapy or radiotherapy. All the samples described above were collected in accordance with the Code of Ethics of the World Medical Association, and informed consent was obtained for experimentation with human subjects. The study was approved by the ethics committee of the local hospital (ethical review report number: 2018-L055).

### Cell Culture

Human GC cell lines (SGC-7901, HGC-27, BGC-823, AGS, and MKN-1) were purchased from the Stem Cell Bank of the Chinese Academy of Sciences (Shanghai, China). Human normal gastric epithelial GES-1 cells were used as the normal control. All cell lines were cultured in RPMI 1640 medium (Corning, Manassas, VA) supplemented with 10% fetal bovine serum (FBS, Gibco, Grand Island, NY), 1% penicillin and streptomycin in a humidified incubator (37°C, 5% CO_2_).

### Nucleoplasm Separation Assay

The nuclear/cytoplasmic RNA was isolated from SGC-7901 cells using a PARIS^™^ Kit (Thermo Fisher Scientific) following the protocol and subjected to qRT-PCR analysis. Up to 10^7^ fresh cultured cells were collected for the experiment. After one wash with phosphate-buffered saline (PBS), cells were resuspended in 300-μl ice-cold cell fractionation buffer, incubated on ice for 5–10 min, and centrifuged at 4°C, 500 × *g*, for 3 min. Then the cytoplasmic fraction was carefully aspirated away from the nuclear pellet. Subsequently, approximately 400-μl ice-cold cell disruption buffer and an equivalent volume of 2× lysis/binding solution were added to the nuclear pellet. After mixing upside down, 400-μl 100% ethanol was added to the mixture. Then the sample mixture was drawn through a filter cartridge. Following orderly washing, centrifugation, and filtration, RNA was eluted twice with elution solution at 95°C. Finally, the isolated nuclear/cytoplasmic RNA was stored at −80°C for later use.

### RNA Exonuclease Digestion Assay

Ribonuclease R (RNase R) was purchased from Geneseed Biotech Co., Ltd (Guangzhou, China). About 3–4 U/μg of RNase R was added to 10-μg total RNA extracted from SGC-7901 and BGC-823 cells. Subsequently, we configured a total of 50-μl digestion reaction system containing 5-μl 10× reaction buffer and then added RNase-free water to make up the total volume. Next, the reaction mixture was incubated at 37°C for 30 min and kept at 70°C for 10 min to inactivate the enzyme before reverse-transcription reaction was performed.

### Total RNA Extraction and qRT-PCR

Total cell and tissue RNA were extracted using TRIzol reagent (Invitrogen, Karlsruhe, Germany), while the peripheral blood samples were pretreated with erythrocyte lysate (Beyotime, Shanghai, China), and then RNA was extracted with TRIzol reagent. Total RNA in each sample was quantified as indicated by NanoDrop^™^ One (Thermo Fisher Scientific, USA). RNA integrity and gDNA contamination were verified by standard denaturalized agarose gel electrophoresis, and purity was determined by spectrophotometry at 260–280 nm. cDNA was synthesized using reverse-transcription reagent (Thermo Fisher Scientific). The relative expression of hsa_circ_0001821 was normalized by the housekeeping gene GAPDH. All primers used in this study were synthesized by RiboBio Corporation (Suzhou, China). The sequences of the target gene are as follows: hsa_circ_0001821: 5′-tggaatgtaagaccccgact-3′ (forward) and 5′-ccatcttgaggggcatcttt-3′ (reverse); PVT1: 5′-gcatggagcttcgttcaagt-3′ (forward) and 5′-gccacagcctcccttaaaac-3′ (reverse); GAPDH: 5′-gaacgggaagctcactgg-3′ (forward) and 5′-gcctgcttcaccaccttct-3′ (reverse). All qRT-PCR assays were performed on the LightCycler 480 system for a total of 20 μl. The 2^−ΔΔCT^ method was used to calculate the relative expression level, and the ΔΔCt value was presented as the  difference between the experimental group (Ct_target_ − Ct_reference_) and the calibrator group (Ct_target_ − Ct_reference_). All experiments were performed independently three times.

### High-Throughput Sequencing

Total RNA was isolated from the tissues using HiPure Total RNA Mini Kit (Magen, Germany). The RNA concentration was determined using the Qubit 3.0 fluorometer (Invitrogen, Carlsbad, CA), and RNA integrity assays were performed using the Agilent 2100 Bioanalyzer (Applied Biosystems, Carlsbad, CA). A RIN value over 7.0 was considered eligible. RNA-seq library was prepared with approximately 2-μg total RNA using KAPA RNA HyperPrep Kit with RiboErase (HMR) for Illumina^®^ (Kapa Biosystems, Inc., Woburn, MA). Briefly, total RNA was incubated at 37°C for 30 min with 10 units RNase R (Epicentre Technologies, Madison, WI) after removal of ribosomal RNA. Next, the RiboMinus RNase R (+) RNA was fragmented, and then first-strand and directional second-strand syntheses were performed. Subsequently, a tailing/adapter ligation approach was performed with the purified cDNA. Finally, the purified, adapter-ligated DNA was amplified. Each library was diluted to 10 nM and pooled equimolar prior to clustering. Paired-end (PE150) sequencing was performed on all samples.

### Identification of Differentially Expressed circRNAs *via* circRNA-Seq

As for the screening of differentially expressed circRNAs, the reads were first mapped to the latest UCSC transcript set using Bowtie 2 version 2.1.0 (Langmead and Salzberg, 2012) and the gene expression level was estimated using RSEM v1.2.15 (Li and Dewey, 2011). Trimmed mean of *M*-value (TMM) was used to normalize the gene expression. Differentially expressed genes were identified using the edgeR program (Robinson et al., 2010). Genes showing altered expression with *P* < 0.05 and more than twofold changes were considered differentially expressed. Uncharacterized circRNAs were regarded as new circRNAs or less studied circRNAs. We firstly used DCC software to identify circRNAs in RNA-seq. The specific procedure is that the DCC software combined with STAR software to compare the sequencing reads to the reference genome, and then the DCC software filtered out the linear sequences aligned to the reference genome. The circRNAs containing the junction site were then identified from the unpaired sequences. After the DCC recognized the circRNA, it was compared to see whether the circRNA_ID (chromosomal coordinates) was in the circBase/circBank database, and if so, the corresponding ID was given; if not, it was represented by NA.

### Construction of the circRNA–miRNA–mRNA Regulatory Network *via* Bioinformatics Software

Based on the circRNA-seq data, we firstly searched for hsa_circ_0001821-targeted miRNAs in the CircInteractome database (https://circinteractome.nia.nih.gov) and found that the context + score percentile of seven miRNAs was greater than 85. Secondly, we searched the miRDB database (http://mirdb.org/miRDB/index.html) for the downstream target genes of the above seven miRNAs and selected the top 10 genes for network mapping.

### Statistical Analysis

The statistical analysis was conducted by GraphPad Prism 7.0 (GraphPad Software, La Jolla, CA) and SPSS 20.0 (SPSS, Inc., Chicago, USA). The clustered heatmap and volcano plots were generated *via* R version 3.5.1 (*R: A Language and Environment for Statistical Computing*, R Core Team, R Foundation for Statistical Computing, Vienna, Austria, 2018, https://www.R-project.org). Student’s *t* test was performed on data of two groups, and paired *t* test was used for comparison of cancerous tissues and adjacent noncancerous tissues. When there were more than two groups of data to compare, we used one-way ANOVA. The ROC curve was established to evaluate the diagnostic value. Youden index (also known as the correct index, Youden index = specificity + sensitivity − 1) was calculated to assess the authenticity of the screening test. The correlation between hsa_circ_0001821 and the clinicopathological parameters was evaluated by chi-square test and Spearman correlation test. A *P* value of less than 0.05 was considered statistically significant.

## Data Availability

The datasets generated for this study can be found in GEO database, GSE131414.

## Ethics Statement

The study was approved by the ethics committee of the Affiliated Hospital of Nantong University.

## Author Contributions

SK wrote the manuscript and performed the experiences; QY helped write the manuscript and perform the experiences; CT helped collect the data; TW interpreted the results; XS and SJ conceived and designed the project, gave vital suggestions and approved the final version.

## Funding

This project was supported by grants from the National Natural Science Foundation of China (81871720).

## Conflict of Interest Statement

The authors declare that the research was conducted in the absence of any commercial or financial relationships that could be construed as a potential conflict of interest.
